# Fatty acids distribution and content in oral squamous cell carcinoma tissue and its adjacent microenvironment

**DOI:** 10.1371/journal.pone.0218246

**Published:** 2019-06-26

**Authors:** Ludmiła Halczy-Kowalik, Arleta Drozd, Ewa Stachowska, Radosław Drozd, Tomasz Żabski, Wenancjusz Domagała

**Affiliations:** 1 Clinic of Maxillofacial Surgery, Pomeranian Medical University, Szczecin, Poland; 2 Department of Biochemistry and Human Nutrition, Pomeranian Medical University, Szczecin, Poland; 3 Department of Immunology, Microbiology and Physiological Chemistry, West Pomeranian University of Technology, Szczecin, Poland; 4 Department of Pathomorphology, Pomeranian Medical University, Szczecin, Poland; University of Illinois, UNITED STATES

## Abstract

Squamous cell carcinoma of the oral cavity mucosa grows under conditions of poor oxygenation and nutrient scarcity. Reprogramming of lipid biosynthesis accompanies tumor growth, but the conditions under which it occurs are not fully understood. The fatty acid content of the serum, tumor tissue and adjacent tumor microenvironment was measured by gas chromatography in 30 patients with squamous cell carcinoma grade 1–3. Twenty-five fatty acids were identified; their frequencies and percentages in each of the environments were assessed. Nineteen of the twenty-five fatty acids were found in tumor tissue, tumor adjacent tissue and blood serum. Of them, 8 were found in all thirty patients. Percentages of C16:0 and C18:1n9 were highest in the tumor, C18:1n9 and C16:0 were highest in tumor adjacent tissue, and C16:0 and C18:0 were highest in blood serum. The frequencies and amounts of C22:1n13, C22:4n6, C22:5n3 and C24:1 in tumor adjacent tissues were higher than those in blood serum, independent of the tumor grade. The correlations between the amount of fatty acid and tumor grade were the strongest in tumor adjacent tissues. The correlations between particular fatty acids were most prevalent for grade 1+2 tumors and were strongest for grade 3 tumors. In the adjacent tumor microenvironment, lipogenesis was controlled by C22:6w3. In blood serum, C18:1trans11 limited the synthesis of long-chain fatty acids. Our research reveals intensive lipid changes in oral cavity SCC adjacent to the tumor microenvironment and blood serum of the patients. Increase in percentage of some of the FAs in the path: blood serum–tumor adjacent microenvironment–tumor, and it is dependent on tumor grade. This dependency is the most visible in the tumor adjacent environment.

## Introduction

Primary squamous cell carcinoma (SCC), which originates in the mucosa of the oral cavity, develops via many non-lethal DNA disturbances in somatic cells that accumulate into a loss of control over cell proliferation, growth and differentiation. During the multiple stages of carcinogenesis, cells develop signals that increase proliferation and growth, prevent cell death and activate angiogenesis, invasion and metastasis. The functions described by Hanahan and Weinberg [1] might be found in the tumor microenvironment (TME) as a result of the reprogramming of energy metabolism and the avoidance of the immune response. Cancer cell development depends on the production of lipids that are necessary for cell membrane formation, protein modification and the transmission of oncogenic signals. Inhibition of lipogenesis by fatty acid synthase (FASN) inhibitors raises the possibility of limiting neoplasm development [2].

De novo lipogenesis in cancer cells, which takes place in the presence of exogenous fatty acids (FAs), has been studied using isotope-labeled exogenous palmitic acid (C16:0) or free FAs in panels of aggressive or non-aggressive human breast cancer, ovarian cancer, prostate cancer, or melanoma cells. Cancer cells take advantage of exogenous acids to promote proliferation and lipid signaling [3]. A general increase in the exogenous FA content causes metabolic alterations that underlie the aggressive behavior of cancer cells. In vitro and in vivo incorporation of exogenous FAs into cancer cells is associated with a redirection of the FAs away from energy metabolism and towards the generation of structural and signaling glycerophospholipids, sphingolipids and other products of lipid metabolism. During oncogenesis, there is a decrease in the creatine phosphokinase (CPK) and oxidative pathways and the use of FAs for energy is inhibited; instead, these FAs are increasingly used as building materials for intensively proliferating cancer cells.

FASN is the enzyme responsible for the endogenous synthesis of saturated FAs from long-chain acetyl-CoA and malonyl-CoA precursors. FASN is overexpressed in many human cancers, such as prostate cancer, breast cancer, bladder cancer, liver cancer, lung cancer and SCC of the oral cavity. Reduced FASN expression occurs simultaneously with decreased SCC proliferation [4].

Guo et al. assessed the synthesis of endogenous FAs with regard to oral cavity mucosa cancer and its surrounding environment: subcutaneous adipose tissue, the sternocleidomastoid muscle, the parotid gland and the submandibular lymph nodes [5]. Based on ^14^C incorporation studies, the FA content was highest in oral cavity mucosa cancers and lowest in muscle tissues. FASN activity in tumor-adjacent tissue was much lower than that in the tumor itself.

Menendez et al. [6] demonstrated an increase in FASN expression at pre-invasive and invasive cancer sites. Increased synthetic FASN expression was preceded by hypoxia, acidification and cell malnutrition at the site. FASN expression was diminished or prevented by disrupting the oncogenic and circulating FAs cascade. The metabolic products of FASN from endogenous FAs participate in cancer evolution by influencing the expression, activity and location of the proteins produced by cancer cells. This process is characteristic of both the transformation of a tumor from benign to malignant as well as tumor progression. An especially important role in carcinogenesis is played by the omega 3 long-chain fatty acids, LA, EPA, DPA and DHA, and omega 6 long-chain fatty acids, AA, DTA and nervonic acid.

Since 1995, the term TME has been used to collectively define cancer cells, stromal cells, various types of mesenchymal cells and the extracellular matrix. The progression of head and neck cancer has been attributed to carcinoma-associated fibroblasts (CAFs) and is associated with increased production of growth factors, cytokines, chemokines, matrix metalloproteinases and inflammatory mediators that enable tumor growth. Therefore, the TME influences the development of cancers and their metastases. The interaction of the TME influences the development of cancers and their metastases. The interaction of the microenvironment and cancer is bidirectional; each interaction partner affects the regulation of gene expression in the other partner or, at least, exerts a selective pressure. Thus, each interaction partner influences the phenotype of the other partner. Cancerous cell interactions with microenvironment components are key indicators during the decision-making process whereby cancerous cells develop into metastases, stay dormant or disappear completely [7,8]. Reflecting the specific features of breast cancer in histologically-normal cancer-adjacent tissue [9] and the prevalence of the wound response signature in histologically normal tissue adjacent to breast cancer [10] inclines for the assessment of this tissue role in cancerogenesis.

To the best of our knowledge, no reports have been published on the quantitative or qualitative relationship of the FAs content in SCC of the oral mucosa, in the adjacent tumor microenvironment (ATME) and in the blood serum. The ATME is the tumor margin, a tissue adjacent to the affected area. Therefore, we sought to assess the FA content in the tumor, ATME and serum of patients with SCC of the oral mucosa and compare the results among these three compartments and with tumor grade. We recorded increase in the frequency and percentage of some of the 25 FAs on the path: blood serum–ATME–tumor. In case of some of the FAs, the increase was dependent on tumor grade. In ATME, the dependency of FAs percentage from tumor grade was the most visible.

## Materials and methods

We obtained the approval of the Bioethical Committee of the Pomeranian Medical University, Szczecin, Poland to conduct the research (Resolution No. KB-0012/01/12 by the Bioethical Committee of Pomeranian Medical University, Szczecin, Poland from 13. February 2012).

### Patients and tissues

We analyzed FAs content in tumor tissue, in the adjacent tumor microenvironment (ATME) and in the blood serum of 30 patients who underwent surgical resections of oral SCC. The research and surgical treatment were performed within the period between March 2012 and March 2013. We provided a standard dietary survey, that is routinely used in patients in Poland before being admitted to the ward. The questionnaire asked whether patients need any special diet (gluten-free, non-dairy, vegan) and whether they apply supplements in their diet. All patients used the usual diet and did not use supplements. Patients taking dietary supplements, patients with coexisting second neoplasm outbreaks, and patients with secondary local neoplasms were excluded from the study. All of the patients participating in the research were informed about the aims and intention of using the results in scientific publications. The authors obtained both oral and written consents from all of the participating patients. All of the patients were adult and aware of the medical procedures on which they were suitably informed. Table 1 shows the clinicopathological details of the 30 patients.

**Table 1:** Characteristics of 30 patients surgically treated due to oral cancer, including tumor data.

**Age**	62,2 ±17,4
**Gender**	10 F, 20 M
**Tumor grade**	**No. of patients**
**G1**	2
**G2**	21
**G3**	7
**Clinical stage**	
T2	7
T3	12
T4	11
**Location**	
Floor of mouth	11
Oral tongue	9
Gums	4
Retromolar trigone	2
Hard palate	2
Buccal mucosa	2

### Chemicals and materials

Chloroform (cat no. 34854), methanol (cat no. 1.06018) and acetic acid were purchased from Merck. Boron trifluoride in methanol (cat no. B1252), NaCl (cat no. S3014) and 2,6-Di-tert-butyl-4-methylphenol (BHT) (cat no. B1378) were supplied by Sigma-Aldrich. Double-distilled water was obtained from a Milli-Q Water System (Millipore, Billerica, MA, USA). Fatty acids standards were bought form Sigma-Aldrich, Neochema (Germany) and Cayman.

### Blood serum and tissue collection

Blood samples were collected from patients into a clean 10 ml polypropylene tube and were allowed to clot at room temperature for up to 2h. After that, the clot were removed by centrifuging at 1,000–2,000 x g for 10 minutes in a refrigerated centrifuge. The separated serum was collected into Eppendorf tubes and stored at—70°C until analysis.

Tumor tissues with healthy tissue margins were fixed in 10% buffered formalin and embedded in paraffin. Sections were stained with hematoxylin and eosin for histopathological diagnosis. In addition, a tumor fragment of approximately 7 mm in diameter and a fragment of a similar size from the macroscopically healthy margins (7 mm from the tumor and 7 mm from the line of incision) were preserved at -70°C for FA extraction. Two pathologists independently assessed the tumor and margin specimens.

### FAME extraction

FAs were extracted based on the protocol published by Folch [11] with minor modifications. For serum samples (0,5 ml) and up to 50mg of the tissue samples, crushed in liquid nitrogen (handheld homogenizer), was added 2,5 ml of Folch mixture (chloroform: methanol: v/v 2:1) and 100 μl of BHT and 100 μl of internal standard C21:0. This mixture was additional mixed by 20 min. by using incubator shaker (New Brunswick Scientific, Excella E24 Series). The samples were then centrifuged at 15 000 rpm for 15 min. (Eppendorf, Centrifuge 5804R). Serum and tissue extract (1 ml) was saponified with 1 ml of 2 M KOH methanol solution at 70°C for 20 min and then methylated with 2 ml of a 14% solution of boron trifluoride in methanol (Sigma-Aldrich, Germany) under the same conditions. Two milliliters of n-hexane and 10 ml of saturated NaCl solution were added. After completely separating the upper (n-hexane phase) and lower layers, the n-hexane phase (1 ml) was collected.

### Gas chromatography (GC) analysis

GC was performed using an Agilent Technologies 7890A GC System equipped with a SUPELCOWAX 10 Capillary GC Column (L × I.D. 15 m × 0.10 mm, df 0.10 μm; Supelco, cat no. 24343). The temperature was increased from 60°C at a rate of 40°C/min up to 160°C; increased at a rate of 30°C/min to 190°C for 0.5 min; increased at a rate of 30°C/min to 230°C for 2.6 min; and maintained for an additional 4.9 min. The total analysis time was 8 min and the gas flow rate was 0.8 ml/min with nitrogen as the carrier gas.

FAs were identified by comparing their retention times with those of highly purified mixture of 25 single standards (Sigma-Aldrich, and Neochema, Cayman Islands) by using ChemStation Software (Agilent Technologies, Cheadle, UK). To control of the fatty acid retention times C21:0 was used as the internal standard. The results are presented as the percentage of the individual fatty acids in the total mass of fatty acids from the examined tissue. These fatty acids are as follows: decanoic acid–C10:0, dodecanoic acid–C12:0, tridecanoic acid–C13:0, tetradecanoic acid–C14:0, myristoleic acid–C14:1, pentadecanoic acid–C15:0, palmitic acid–C16:0, palmitoleic acid–C16:1, heptadecanoic acid–C17:0, stearic acid–C18:0, oleic acid–C18:1n9 (OA), vaccenic acid–C18:1trans11 (VA), linoleic acid–C18:2n6 (LA), gamma linolenic acid–C18:3n6 (GLA), α linolenic acid–C18:3n3 (ALA), stearidonic acid–C18:4, arachidonic acid–C20:4 (ARA), eicosapentaenoic acid–C20:5 (EPA), behenic acid–C22:0, erucic acid–C22:1n13, tricosanoic acid–C23:0, docosatetraenoic acid–C22:4n6 (DTA), docosapentaenoic acid–C22:5n3 (DPA), docosahexaenoic acid–C22:6n3 (DHA), nervonic acid–C24:1.

The fatty acid content was calculated as follows: sum of the omega3 fatty acids (n3) = C18:3n3+C18:4+C20:5+C22:5n3+C22:6n3; sum of the omega6 fatty acids (n6) = C18:2n6+C18:3n6+C20:4+C22:4n6; saturated fatty acids (**SFAs**) = C8:0+C10:0+C12:0+C14:0+C15:0+C16:0+C17:0+C18:0+C22:0+C23:0+C26:0); monounsaturated fatty acids (**MUFAs**) = C14:1+16:1+C18:1n9+C18:1trans11+C22:1n13 +C24:1, poly-unsaturated fatty acids (**PUFAs**) = 18:2n6+C18:3n6+C18:3n3+C18:4+C20:4+C20:5+C22:4n6+C22:5n3+C22:6n3. Unsaturated fatty acids **UFAs** were the sum of the MUFAs and PUFAs.

### Statistical analyses

All continuous variables were evaluated for the normality of distribution using the Kolmogorov-Smirnov test. The continuous variables were described by means, standard deviations, medians, quartiles and minimum and maximum values. Differences between two groups were analyzed using Student’s t-test or the Mann-Whitney U test. For multiple-group comparisons, analyses of variance (ANOVAs) and/or Kruskal-Wallis tests were performed. Differences of p < 0.05 were considered significant for all of the performed tests.

All statistical analyses were performed using STATA 11 software (2009). Significant correlations between the analyzed fatty acids were described according to the scheme proposed by Dallal [12].

Correlation analysis and the illustration of Pearson’s coefficients.

Pearson’s correlation coefficients were calculated on the correlation analysis. The R package Corrgram was used to display the correlations between the analyzed parameters as a correlogram or a coefficient eigenvector plot43. A correlogram is a direct visual display of the matrix of Pearson’s coefficients.

### Correlogram analyses

Correlograms were plotted for the analyzed data using the Corrgram R package (http://cran.r-project.org/web/packages/corrgram/index.html) [13]. Pearson’s correlation coefficients were calculated using the cor.test function in r and p-values. Significant correlations with p-values ≤ 0.05 were considered significant and were visualized as red or blue dots on the correlogram, depending on the direction of the correlation. The dot size in the correlation matrix is proportional to the strength of the correlation.

## Results

### Frequency of fatty acids in tumor, ATME and blood serum

We identified 25 FAs in 30 patients treated surgically due to oral squamous cell carcinoma. Only 8 of these FAs (C14:0, C16:0, C16:1, C18:0, C18:1n9, C18:2n6, C20:4, C22:6n3) were found in all three environments, i.e. in tumor, ATME and blood serum, in 30 of the patients, i.e. in 100% of all cases. In 29 of the patients 1 FA (C13:0) was recorded only in blood serum. Another 11 FAs were found in tumor, ATME, and in serum in various percentage of the patients. Furthermore, 5 FAs, not present in serum, were found in tumor and ATME in various percentage of the patients.

Higher frequency in tumor than in ATME was recorded for 6 FAs (C15:0, C17:0, C18:3n6, C20:5, C22:1n13, C24:1) and frequency of three FAs (C14:1, C18:1t11, C18:3n3) decreased in tumor in comparison with ATME. The most significant decrease was recorded for FA C18:3n3 (60% vs. 87%).

Altogether 9 FAs were found more often in ATME than in serum. The greatest increase in frequency in ATME in comparison with serum was noted for the following FAs: C24:1, C14:1, C22:4n6, C22:5n3 (93% *vs*. 0%, 100% *vs*. 10%, 90% *vs*. 3%, 100% *vs*.20% respectively). By contrast, frequency of 6 FAs (C12:0, C13:0, C15:0, C17:0, C18:1t11, C18:3n6) decreased in ATME as compared to serum. The most significant decrease was recorded for C13:0 (0% *vs*.97%).

Lack of some FAs in serum, increase of other FAs frequency in ATME in comparison with serum, and decrease in frequency of some other FAs in tumor in comparison with ATME point to ATME as the environment which is extremely active in lipid metabolism which accompanies neoplasia. Table 2 shows the data on frequency of all indentified FAs.

### Fatty acids content percentage in tumor, ATME and serum

Medium chain and long chain FAs with the shortest chains, i.e. C10:0, C12:0, C13:0, C14:1, C15:0 constituted most often less than 1% of FAs detected in the environments studied. FA C16:0 accounted for the greatest percentage in tumor (27%), C18:1n9 in ATME (35%) and C16:0 in serum (37%).

Increased percentage of FAs in tumor in comparison with ATME was recorded for 11 FAs, and the greatest one involved C24:1 (1.31% *vs*. 0.36%). Percentage of 11 FAs was higher in ATME than in serum and the biggest difference was seen for C22:4n6 (0.39% *vs*. 0.01%).

Decrease in FAs percentage in tumor in comparison with ATME was detected for 6 FAs with the biggest one involving C18:3n3 (0,27% *vs*. 0,69%). Decrease in FAs percentage in ATME in comparison with serum was found for 11 FAs. The biggest difference concerned C18:3n6 (0,01% *vs*. 0,39%).

Increase in percentage from serum through ATME to tumor was recorded for 2 long-chain FAs: C22:4n6 and C22:5n3. The increase was greater in case of C22:4n6 (from 0.01%, through 0.39%, to 1.10%) than C22:5n3 (from 0.10%, through 0.57%, to 1.02%).

Table 2 shows the data on percentage of FAs content in tumor, ATME and serum.

**Table 2:** Frequency of fatty acids (%) and percentage of fatty acids content (%) in tumor, ATME, and blood serum.

Fatty acid	Frequency	Mean %	Frequency	Mean %	Frequency	Mean %	Differences
	Tumor		ATME		Blood serum		
**C10:0**	53	0.58±0.7	50	0.18±0.5	60	0.15±0.1	1,2,3
**C 12:0**	63	0.13±0.2	73	0.18±0.2	100	0.41±0.2	2,3
**C13:0**	0	0±0.0	0	0±0.0	97	0.12±0.0	2,3
**C14:0**	100	1.99±2.1	100	2.12±1.6	100	1.39±0.3	1,2
**C14:1**	80	0.17±0.2	100	0.27±0.2	10	0.01±0.0	1,2,3
**C15:0**	100	0.38±0.2	93	0.27±0.2	100	1.10±0.3	1,2,3
**C16:0**	100	26.86±4.8	100	25.55±5.9	100	37.26±2.1	2,3
**C16:1**	100	3.03±1.6	100	5.43±3.0	100	1.48±0.7	1,2,3
**C17:0**	100	0.42±0.1	80	0.28±0.3	100	0.54±0.1	1,2,3
**C18:0**	100	18.61±9.3	100	11.21±10.6	100	20.03±3.0	1,2,3
**C18:1n9**	100	21.40±9.7	100	35.03±13.9	100	16.56±3.1	1,2,3
**C18:1t11**	77	3.01±2.0	83	3.05±1.6	100	1.74±0.3	2,3
**C18:2n6**	100	9.47±3.0	100	9.96±2.8	100	12.09±2.0	2,3
**C18:3n6**	17	0.03±0.1	10	0.01±0.0	93	0.39±0.1	1,3
**C18:3n3**	60	0.27±0.3	87	0.69±0.5	83	0.43±0.3	1,2,3
**C18:4**	13	0.03±0.1	17	0.04±0.1	0	0±0.0	1,2,3
**C20:4**	100	7.04±3.1	100	2.66±2.5	100	4.21±0.8	1,2,3
**C20:5**	100	0.30±0.2	90	0.17±0.1	80	0.47±0.3	1,2,3
**C22:0**	77	0.29±0.3	57	0.15±0.2	0	0±0.0	2,3
**C22:1n13**	100	0.27±0.2	80	0.17±0.3	0	0±0.0	1,2,3
**C23:0**	57	0.55±0.6	57	0.48±0.6	0	0±0.0	2,3
**C22:4n6**	90	1.10±1.1	90	0.39±0.3	3	0.01±0.1	1,2,3
**C22:5n3**	100	1.02±0.4	100	0.57±0.2	20	0.10±0.2	1,2,3
**C22:6n3**	100	1.70±0.7	100	0.78±0.6	100	1.52±0.4	1,3
**C24:1**	97	1.31±1.5	93	0.36±0. 6	0	0±0.0	1,2,3

1—difference between tumor and ATME

2—difference between tumor and blood serum

3—difference between ATME and blood serum

P for all differences < 0.05, except for C10 and C18:4 where differences were not significant

### Percentage of content of FAs groups: SFA, UFA, MUFA, PUFA, series n6 and n3 of PUFA and ratios C18:2n6/C18:3n3, C20:4/(C20:5+C22:6n3) in tumor, ATME and blood serum

Percentage of UFA was the highest (>50%) among the assessed FAs in ATME and tumor. PUFAs constituted about 1/5 of all FAs in tumor and serum. The ratio n6/n3 was significantly lower in tumor than in serum (5,51 *vs*. 7,18). The ratio C20:4/ C20:5+C22:6n3 was the lowest in serum, and the highest in tumor. Table 3 contains detailed information on percentages of content of particular FAs groups in three environments examined.

**Table 3:** Percentage of content of FAs groups: SFA, UFA, MUFA, PUFA, series n6 and n3 of PUFA and ratios C18:2n6/C18:3n3, C20:4/(C20:5+C22:6n3) in tumor, ATME and blood serum.

FAs groups	Tumor mean %	ATME mean %	Serum mean %	Differences
**SFA**	**49.86±12.8**	**40.44±15.5**	**60.99±5.2**	**1,2,3,**
**MUFA**	**29.18±11.7**	**44.31±16.7**	**19.79±3.9**	**1,2,3**
**PUFA**	**20.95±6.0**	**15.26±3.7**	**19.22±3.0**	**1,2,3,**
**n6**	**17.63±5.1**	**13.01±3.1**	**16.70±2.5**	**1,2,3**
**n3**	**3.32±1.2**	**2.24±0.8**	**2.52±0.8**	**1,3**
**n6/n3**	**5.51±1.3**	**6.21±1.6**	**7.18±2.0**	**1,2**
**C18:2n6 /C18:3n3**	**17.48±19.2**	**15.91±15.2**	**22.81±14.0**	**2**
**C20:4/(C20:5+C22:6n3)**	**3.55±1.0**	**2.58±0.9**	**2.28±0.7**	**3**
**UFA**	**50.14±12.8**	**59.56±15.5**	**39.01±5.2**	**1,2**
**PUFA/SFA**	**0.45±0.2**	**0.42±0.1**	**0.32±0.1**	**2,3**
**MUFA/SFA**	**0.68±0.4**	**1.37±0.8**	**0.33±0.1**	**1,2,3**
**UFA/SFA**	**1.13±0.5**	**1.78±0.9**	**0.65±0.1**	**1,2,3**

1—difference between tumor and ATME

2—difference between tumor and blood serum

3—difference between ATME and blood serum

P for all differences < 0.05

### Percentage of FAs content in tumor, ATME and blood serum *vs*. tumor grade

Percentage of content of only 11 FAs in tumor, ATME and serum showed statistically significant differences between tumors G1+G2 vs. G3 (p<0.05). The following FAs constituted higher percentage of total FAs content in grade 3 than in grade 1+2 tumors: C23:0 in tumor and ATME, C10:0 and C20:5 in serum, and C18:0, C22:0, C22:1n13, C22:6n3 in ATME. The following FAs constituted lower percentage of total FAs content in grade 3 than in grade 1+2 tumors: C10:0 and C12:0 in tumor and ATME, C18:1n9, C18:2n6, C18:3n3 in ATME. Detailed data on content percentages of particular FAs in tumor, ATME and serum with regard to tumor grade is supplied in S1 Table.

In summary, analysis of these differences showed following associations. In tumor, percentage of C23:0 correlated positively and percentages of C10:0 and C12:0 correlated negatively with tumor grade. In ATME, percentages of C18:0, C22:0, C22:1n13, C23:0, and C22:6n3 correlated positively with tumor grade, however percentages of C10:0, C12:0, C18:1n9, C18:2n6, and C18:3n3 correlated negatively with tumor grade. In serum percentages of C10:0 and C20:5 correlated positively with tumor grade. Relation between percentage of C10:0 and tumor grade depended on the environment: being positive in serum and negative in ATME and tumor. ATME was the environment in which the greatest number of FAs (i.e. 10 of 11) showed differences in percentage content between G1+2 and G3 tumors and these differences were the largest.

### Percentage of content of FAs groups: SFA, UFA, MUFA, PUFA, series n6 and n3 of PUFA and ratios C18:2n6/C18:3n3, C20:4/(C20:5+C22:6n3) in tumor, ATME and serum vs. tumor grade

Of all FAs groups studied in tumor tissue there was only a negative correlation between C20:4/(C20:5+C22:6n3) ratio and tumor grade (the ratio lower in grade 3 than in grade 1+2 tumors). In ATME there was a positive correlation between percentage of SFA content and tumor grade and negative correlations between percentage of content of UFA, MUFA, rates of PUFA/SFA, MUFA/SFA, UFA/SFA and tumor grade. High tumor grade was associated with higher percentage of SFA and with lower percentage of UFA in ATME. In serum we found only a positive correlation between percentage of n3 PUFAs content and a negative correlation between n6/n3 ratio and tumor grade. High grade was associated with higher percentage of n3 PUFAs and lower percentage of n6 PUFAs in serum. S2 Table presents detailed data on the analyzed associations.

### Percentage ratio of particular FAs in tumor/ATME, ATME/serum and in tumor/serum vs. tumor grade

The ratios of percentage contents of particular FAs in tumor/ATME, ATME/serum and in tumor/serum were calculated. Tumor/ATME, ATME/serum and tumor/serum ratios lower than 1 were observed for FAs C12:0, C13:0 and C18:4 irrespective of tumor grade. Similarly, ATME/serum and tumor/serum ratios were lower than 1 for FAs C15:0, C16:0, C17:0, C18:3n6, C20:5, C22:0, C22:1n13 and C23 irrespective of tumor grade.

Tumors G1+G2 exhibited tumor/ATME rations higher than 1 for 16 FAs, ATME/serum–for 9 FAs and tumor/serum for 10 FAs whereas in tumors G3 tumor/ATME rations were higher than 1 for 11 FAs, ATME/serum–for 7 FAs and tumor/serum for 9 FAs. This means that percentage content of 16 FAs in tumors G1+G2 and 11 FAs in tumors G3 was increased in tumor tissue as compared to ATME respectively. Furthermore, percentage content of 9 FAs in tumors G1+G2 and 7 FAs in tumors G3 was increased in ATME as compared to serum respectively. Finally, percentage content of 10 FAs in tumors G1+G2 and 9 FAs in tumors G3 was increased in tumor as compared to serum respectively.

This growth of particular FAs percentage between serum and ATME, ATME and tumor was recorded for the majority of FAs, irrespective of tumor grade. Statistically significant, positive association of tumor grade with ATME/serum ratio of FA percentage content was recorded for C17:0 (p = 0.0462) and C18:0 (p = 0.0133), with tumor/serum ratio for FA C20:5 (p = 0.0194), and with tumor/ATME ratio for FA C23:0 (p = 0.0003). Statistically significant, negative association of grade with tumor/ATME ratio was observed for C10:0 (p = 0.0091), with each of the calculated ratios [tumor/ATME (p = 0.0061), ATME/serum (p = 0.0024), tumor/serum ratios (p = 0.0028)] for C12:0, and with tumor/ATME ratio for C18:0 (p = 0.0226). Detailed data on percentage ratio of content of particular FAs in tumor/ATME, ATME/ serum and in tumor/serum vs. tumor grade is supplied in S3 Table.

### Correlations of FAs percentage in tumor, ATME and blood serum including tumor grade

**Fig 1:**
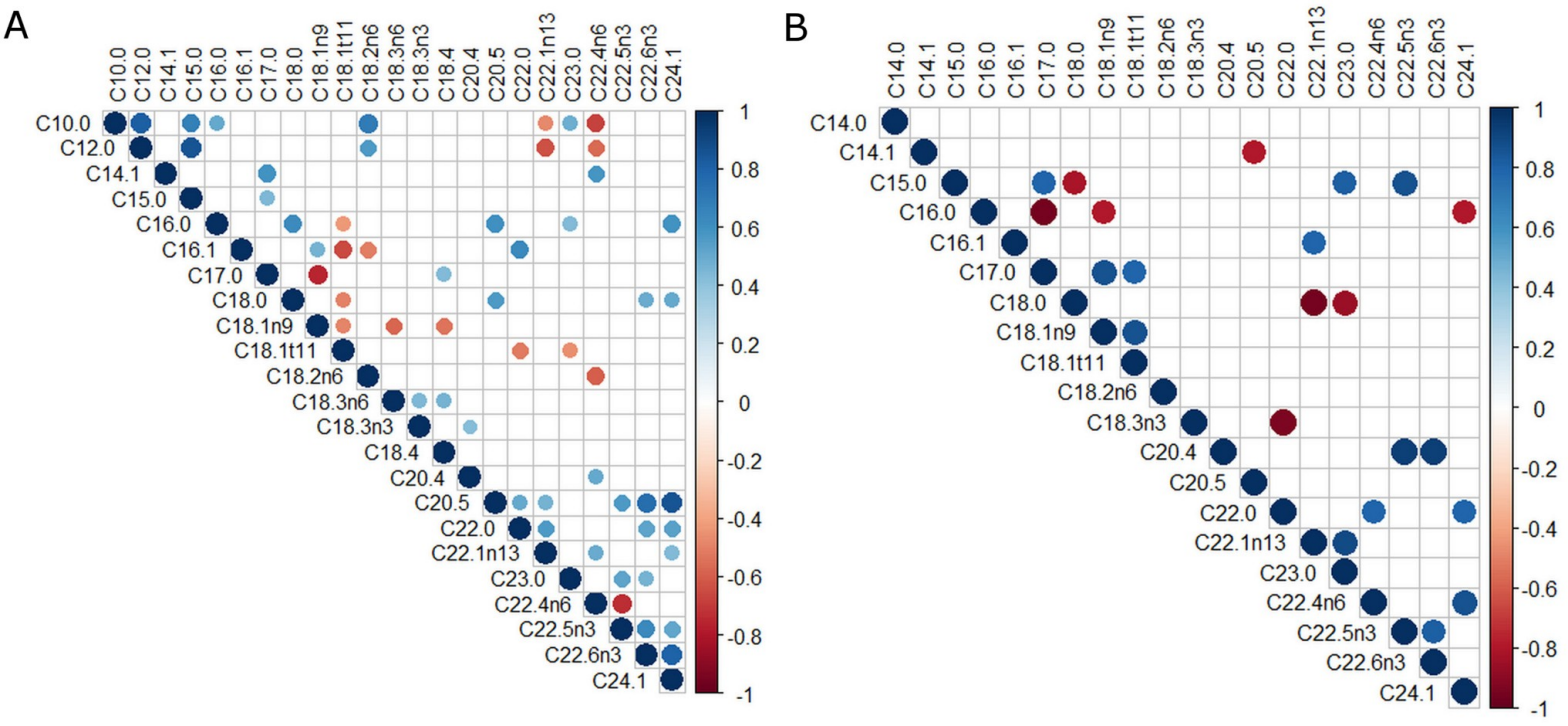
Correlations of FAs percentage in tumor. A. SCC grade 1+2. B. SCC grade 3. Red or blue dots on the correlogram, depending on the direction of the correlation, the dot size in the correlation matrix is proportional to the strength of the correlation.

**Fig 2:**
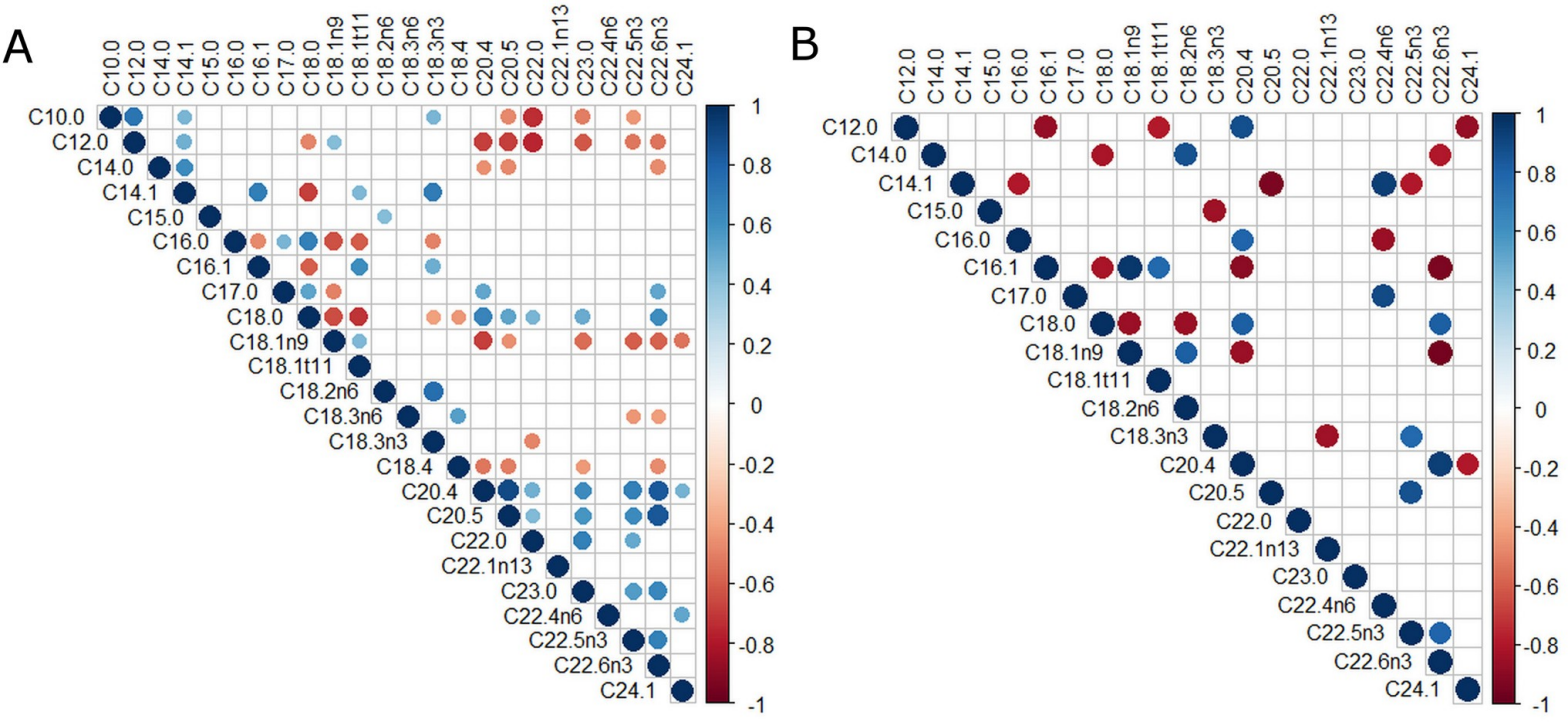
Correlations of FAs percentage in ATME. A. SCC grade 1+2. B. SCC grade 3. Red or blue dots on the correlogram, depending on the direction of the correlation, the dot size in the correlation matrix is proportional to the strength of the correlation.

**Fig 3:**
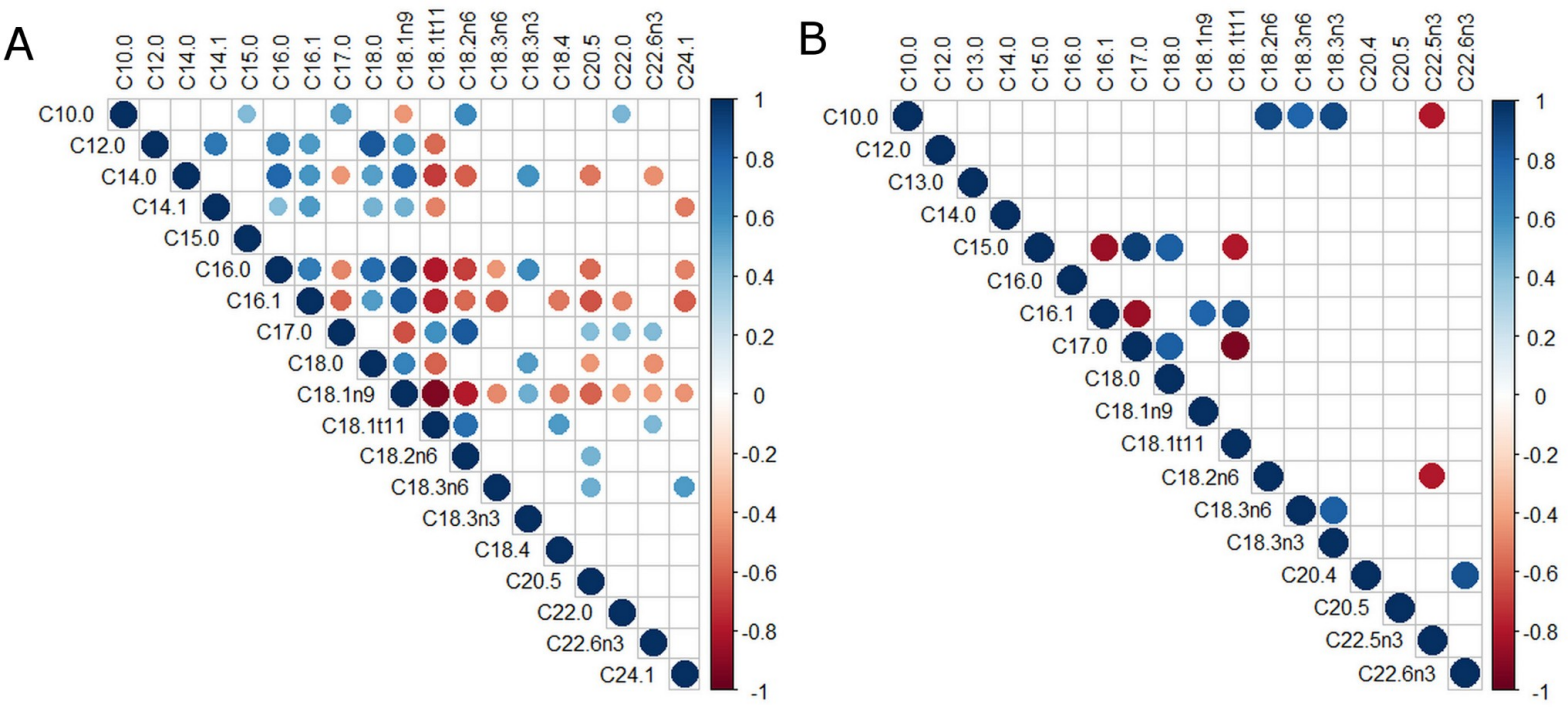
Correlations of FAs percentage in blood serum. A. SCC grade 1+2. B. SCC grade 3. Red or blue dots on the correlogram, depending on the direction of the correlation, the dot size in the correlation matrix is proportional to the strength of the correlation.

Correlations between percentage of FAs in tumor, ATME and serum including tumor grade were found and compared. Only statistically significant (p < 0.05), strong, and very strong correlations (0.7 < r < 0.9) were considered. Figs 1A, 1B, 2A, 2B, 3A and 3B show all of the statistically significant correlations. Correlations between percentage content of particular FAs within the same environment differed depending on the grade of the tumor.

In tumor tissue, there were 7 strong mutual FAs correlations recorded for grade 1+2 tumors, and 22 strong and very strong mutual FAs correlations for grade 3 tumors. Among the strongest positive correlations in grade 3 tumor tissue were the following: C20:4 with C22:5n3 (r = 0.93) and C20:4 with C22:6n3 (r = 0.93), whereas the strongest negative correlation was the one between C22:0 and C18:3n3 (r = -0.93). The strongest correlation in tumor grade 1+2 was a positive correlation between C24:1 and C20:5 (r = 0.85). Fig 1A and 1B show all statistically significant (p <0.05) correlations between percentages of FAs in tumor.

In ATME there were 8 strong mutual FAs correlations observed in grade 1+2 tumors and 33 strong and very strong mutual FAs correlations in tumors grade 3. Within grade 3 tumors the strongest positive correlation in ATME was detected between C16:1 and C18:1n9 (r = 0.96) and the strongest negative correlation between C18:1n9 and C22:6n3 (r = -0.96). The strongest correlation in ATME within grade 1+2 tumors was a positive correlation between C20:4 and C20:5 (r = 0.89). Fig 2A and 2B show all statistically significant (p <0.05) correlations between percentages of FAs in ATME.

In blood serum of patients with grade 1+2 tumors, there were 14 strong mutual FAs correlations and with grade 3 tumors– 16 strong or very strong mutual FAs correlations. The strongest positive correlation was correlation between C15:0 and C17:0 (r = 0.93); The strongest negative correlation was correlation between C17:0 and C18:1trans11 (r = -0.93). The aforementioned correlations accompanied tumor grade 3. The strongest correlation in serum for tumor grade 1+2 was a negative correlation between C18:1n9 and C18:1trans 11 (r =-0.91). Fig 3A and 3B show all statistically significant (p <0.05) correlations between percentages of FAs in blood serum.

All of the statistically significant correlations are indicative of close relations between particular FAs. The number of strong and very strong mutual correlations between FAs in tumor and ATME was higher in grade 3 than grade 1+2 tumors. The correlations indicate that in tumors grade 3 lipogenesis in tumor tissue coexisted with inflammation and in tumors grade 1+2 lipogenesis was modulated by EPA. The correlations found in ATME adjacent to tumor grade 3 suggest the dependence of FA C18:1n9 percentage (major FA in this environment) on supply in diet and natural limitation of this FA by DHA (tumor grade 3), and in tumor grade 1+2 accumulation of EPA as an answer to inflammation. In blood serum, the calculated correlations suggest C18:1trans11 as a FA limiting the frequency and percentage of the following FAs: C15:0 and C17:0 (in tumor grade 3), as well as FA C18:1n9 (in tumor grade 1+2).

## Discussion

Changes to lipid organization that result in cancer initiation and progression contribute to the understanding of carcinogenesis and identification of potential therapeutic targets. The activity of enzymes that participate in the synthesis and endogenous changes of fatty acids, probably created for a fast growing tumor, is higher in the tumor than in healthy tissues. The increased de novo synthesis of fatty acids is a feature of intensive tumor growth [5].

Lipid metabolism in rapidly proliferating cancerous cells is based on redirecting the carbon from energy production into membrane biosynthesis and signal particles. Phosphatidylcholine, phosphatidylethanolamines, sterols, sphingolipids and lysophospholipids make up the majority of cell membrane lipids. All of them originate with acetyl-CoA and many of them contain fatty acids that originate with external sources or in de novo synthesis. While healthy human cells use exogenous fatty tissues, tumor cells synthesize de novo fatty acids. Decreasing the accessibility of fatty acids through the use of chemical inhibitors of lipid enzymes slows down the growth of some cancers [14].

Increased expression of lipogenic enzymes, such as acetyl-CoA-carboxylase (ACC), fatty acid synthase (FASN) and ATP citrate lyase (ACLY), accompanies the phenotypic changes in the majority of tumors. The overexpression of FASN forecasts a poor prognosis in cancer patients [15].

### Redistribution of individual FAs in tumor, ATME and in blood serum

In the margin free of tumor invasion (macroscopically and microscopically) the amount of C18:1 was higher than that of C18:0. This finding suggests the overexpression of FASN. We did not measure FASN expression directly; instead we used an increase in the percentage of C18:1 in the ATME as indirect evidence for FASN expression. Interestingly, C18:1 content in the serum was relatively low, which supports the theory of increased acid synthesis in the microenvironment (rather than obtaining fatty acids from the circulation). A site which has not been transformed is tuned into carcinogenesis because it produces fatty acids associated with the promotion of tumors.

Among the 25 FAs, there were three, i.e. C16:0, C18:0, C18:1n9, which were found in 100% of the patients and represent the greatest percentage in the researched environments of tumor, ATME and serum. The percentage of C16:0 and C18:0 in ATME in comparison with serum was significantly lower, however, it was greater in tumor than in ATME. The percentage of C18:1n9 was greater in ATME than in tumor or serum.

It is probable that tumor growth is associated with another metabolism of the FAs in tissues adhered than in healthy tissues and it can indicate to the increased activity of FASNs as well as stearoyl-CoA desaturates (SCDs) [16, 17].

It has been shown that a microenvironment rich in oleic acid supports gastric cancer progression and migration of cancer cells, through the induction of AMP-activated protein kinase (AMPK) by oleic acid [18].

We have found that microenvironment adjacent to SCC was also enriched with oleic acid (C18:1n9) and in addition with other FAs such as C14:1, C16:1, C18:3n3, C22:4n6, C22:5n3 and C24:1. This finding suggests that oleic acid (and some other FAs) may have a role in progression of SCC of oral cavity.

The percentage of FAs C18:1n9 and C18:3n3 contained in tumor was lower in comparison with the content of FAs in healthy oral cavity mucosa; the percentage of C18:0 was greater than the norm [19].

Our findings concerning oleic (C18:1n9), linoleic (C18:2n-6), and stearic (C18:0) acids percentages in SCC *vs*. ATME are in partial consent with those of Askari et al [20] who reported that oleic acid and linoleic acid levels in oral cancer tissues were significantly lower than those in healthy tissues and that stearic acid levels were highest in tumor tissues. In our findings the stearic acid level was third in volume in the tumor after palmitic acid and oleic acid.

Redistribution of particular FAs between the serum and the ATME and between the ATME and the tumor are interesting. Among 8 FAs found in all of the examined environments (tumor, ATME and serum) in all of the patients, percentage of 3 FAs: C14:0, V16:1, C18:1n9 was significantly higher in ATME than in tumor and serum. Percentage of 2 other FAs: C18:0 and C20:4 was significantly higher in tumor than in ATME. Oleic acid (C18:1n9) was the FA of which the highest percentage in ATME, was the highest percentage among all isolated FAs, and it was lower in tumor than in ATME.

We wanted to know why the levels of endogenous fatty acids were higher in the ATME than in the blood serum. The early activation of fatty acid synthase compensates for the lack of oxygen and exogenous fatty acids associated with a lack of angiogenesis [21]. This amazing metabolic situation serves the high proliferation rate of the cells that survive the absence of exogenous fatty acids. Whether the balance between particular exogenous fatty acids and fatty acid synthase producing endogenous fatty acids decides the development and malignancy of a neoplastic change was investigated by Menendez et al. who found that: ‘A recent identification of cross-talk between FASN and well-established cancer-controlling networks begins to delineate the oncogenic nature of FASN-driven lipogenesis’ [22]. These data show that FASN-dependent endogenous lipogenesis controls the metastatic phenotype promoted by cysteine-rich 61, (Cyr61/CCN1) [23].

Here, the presence of 5 FAs (i.e., C18:4, C22:0, C22:1n13, C23:0, and C24:1) in the ATME that had levels below detection limit in the blood serum is of note. The content of the FAs (except for C18:4) in the TU was higher than in the ATME. In the ATME, the content of 4 FAs (i.e., C18:3n3 GLA, C20:5 EPA, C22:4n6 DTA, and C22:5n3 DPA) was higher than in the BS with the exception of C18:3n3, which was higher in the BS than in the TU. These data suggest that the ATME creates a favorable environment for the generating FAs not supplied by the BS and for increasing the amounts of FAs supplied by the BS that are needed for growing the tumor.

### DPA and DTA in tumor, ATME and serum

The levels of C22:5n3 (DPA) increased from the serum, through the ATME to the tumor independent of tumor grade. The values of all the assessed ratios (tumor/ATME, ATME/serum, and tumor/serum) were greater than 1. C22:5n3 correlated positively with C10:0 in the serum when the tumor grade was 3 and it correlated positively with C10:0 and C12:0 in the ATME when the tumor grade was 1+2.

Docosapentaenoic acid (DPA) is rarely the main subject of a research study; it is usually presented as an agent between eicosapentaenoic acid (EPA) and docosahexaenoic acid (DHA) in the n-3 PUFA synthesis pathway. DPA is an important biologically active anti-inflammatory fatty acid that reduces the aggregation of platelets and improves the lipid profile of the blood serum [24]. Sprecher, Berquin and Artenburn [25–27] emphasize the role of DPA in lipid transitions in mammals that cannot synthesize de novo n3 and n6 PUFAs. According to Yazdi [28], DPA may act as a reserve for the majority of n-3 fatty acids. DPA is a strong inhibitor of angiogenesis and carcinogenesis because it suppresses VEGF expression (vascular endothelium growth factor) [29]. Anti-proliferative and pro-apoptotic effect of DPA on colorectal carcinoma cells has been shown in vitro [30].

Despite of expected anticancer effect, independent from tumor grade increase of DPA percentage, in serum, through ATME to tumor, and especially dependent from tumor grade negative correlations with FAs which are a source of energy, mainly in pathologic conditions such as inflammation which accompanies cancer [31, 32], should incline to recognize this FA as an actively involved in lipid changes that accompany the tumor.

Our results show DTA as a FA present in serum very rarely and in minor quantities. Its frequency and percentage increased from serum through ATME up to tumor, independently from tumor grade. In the tumor, DTA correlated positively with C22:0; in the ATME–with C14:1.

DTA, also known as adrenic acid, is created through the elongation of arachidonic acid chain, or chain elongation and desaturation of essential fatty acid—linoleic acid. DTA is present in adrenal glands, brain, kidneys, and blood vessels. Its metabolites regulate blood flow in adrenal glands blood vessels [33].

DTA has not been studied in the SCC of oral cavity. Ghebremeskel [33] emphasized the increase of DTA level and increase of DPA/DTA ratio in serum of Korean pregnant women, suggesting that the increase is a response to mobilization of ARA and DHA from mothers’ red blood cells membrane to meet the growing fetal needs.

Positive mutual correlations of DTA and C14:1 in ATME estimated by our team may be caused by significantly higher percentage of C14:1 in ATME than in serum, as it may happen in response to tissues malnutrition and beta-oxidation [34]. Similar correlations with C22:0, FA which increases cholesterol level [35], may be associated with lowered cholesterol level which accompanies cancer [36]. DTA may be the one FA which contributes to supplementation of FAs deficiencies caused by tumor growth.

### Tumor grade vs. individual FAs and FAs groups

We observed differences in the percentages of fatty acids in the tumor, ATME and serum that were dependent on tumor grade. The most numerous correlations were found in ATME; among the positive ones, there was a correlation with DHA, among the negative ones, there were correlations with: C18:1n9, C18:2n6 and C18:3n3. Less numerous correlations were observed in tumor, and they involved C10:0 and C12:0—negative correlation, C23:0—positive correlation. In the serum, C10:0 and EPA positively correlated with tumor grade. Thought-provoking are positive correlations with DHA and EPA, which influence is assessed as potentially anticancer [37, 38, 39], and negative ones with C18:1n9, C18:2n6 (FAs which may increase tumor growth [40]) with tumor grade. Reports on tumor grade influence on lipids level indicate decreased level of lipids in serum, independently on tumor grade [41], or mention statistically non-significant dependence on tumor grade [42–45].

Groups of FAs, MUFAs and PUFAs found by our research team in blood serum represented the lesser percentage than in healthy patients [46], however, SFAs were reported in our patients in greater percentage.

We have established that the greatest differences in FAs groups were present in ATME, and they were dependent on tumor grade. UFAs and MUFAs were negatively correlated with tumor grade, whereas the SFAs were positively correlated with tumor grade. Zhao [47] assessing data from epidemiological meta-analysis of endometrial cancer risk formulated similar statements. We have calculated that n3 FAs in the serum were positively correlated with tumor grade, the n6/n3 ratios in the serum were negatively correlated with tumor grade, and that C20:4/(C20:5+C22:6n3) ratio in the tumor was negatively correlated with tumor grade.

We were surprised by a positive correlation of n3 FAs with tumor grade, because these FAs are perceived more often as anticancer or lowering cancer risk [48]. According to Mansara [49], an increase intake n3 FAs supply may help prevent and/or cure cancer; however, the variation in the n6/n3 FA ratio influences the modulation of signals that can regulate cell growth. Engagement of particular FAs and FAs groups in lipid changes accompanying prostate and breast cancer growth is predictable [50–52] however, it is sometimes different from the expected.

Observations by Zhang et al. [53] concerning FAs in serum in the patients treated due to polyps and cancer of large intestine, on positive correlations of n-3 PUFAs, C22:6 n-3, total n-6 PUFAs, C18:2 n-6, ratio C20:4 n-6 and (C20:5 n-3 +C22:6 n-3) with tumor, and on negative correlations of these FAs and C20:4 n-6 and (C20:5 n-3 +C22:6 n-3) ratio with polyps led the Authors to the conclusion that n3 FAs played a controversial role both in polyps creation and carcinogenesis.

Juxtaposition of the data on n3 PUFAs contribution to carcinogenesis allowed Baracos [54] to present a thesis that n-3 PUFA have an influence on cancer at all stages of initiation, promotion, progression, and neoplastic transformation. It is possible thanks to n3 PUFAs ability to change the balance between pro- and antiapoptotic activity of Bcl-2 protein in favor of cancer cell death [55], thanks to EPA ability to suppress cell division through translation initiation suppression [56], thanks to the mechanisms of attenuation of wasting by *n*-3 PUFA centre largely around catabolic signal transduction by cytokines, eicosanoids and tumour-derived proteolysis-inducing factor [57], through antiangiogenic [58] and antimetastatic [59] activity of n3 PUFAs. In patients with advanced cancer thanks to n3 PUFAs supplementation it was possible to suppress pro-inflammatory response accompanying cancer, and obtain body mass gain positively correlating with EPA level in serum [60].

Accordingly to our beliefs, n3 PUFAs participate in cancerogenesis through decrease of inflammation in tumor, lipogenesis in ATME limiting, and egzogenic FAs in serum supply limitation.

### Mutual correlations between individual FAs

Correlations estimated by our research team between the percentage of FAs in tumor, ATME and serum indicated to the presence of distinct associations for each of the environments, in addition, dependent on tumor grade. Whereas the strongest correlations were observed in tumor grade 3, they could be assigned partly to the small number of the researched patients. However, in comparison to grade 1+2, the correlations concerned lesser number of the FAs. The results along with scientific literature data enabled to create the image of changes accompanying the tumor which are present in the researched environments.

In the existing tumor with accompanying inflammation the strongest correlations showed support for the inflamed condition [61–64], decrease in energy production [32, 65], as well as pro-apoptotic effect limitation [66]. Our research team did not research the condition of inflammation; it only can be concluded on the basis of the presence or increased number of pro-inflammatory FAs which are the precursors of pro-inflammatory eicosanoids, prostaglandins and tromboxanes.

In ATME, microenvironment adhered to the tumor, macroscopically and microscopically free from cancerous infiltration, correlations between FAs showed the possibilities of suppressing the progression of the already existing cancer promotion [67–69], limitation of inter-cellular communication and energy production [65], initiation of the response to progressing inflammation in tumor [70–75].

In blood serum, on the one hand there were the correlations between FAs concerning FAs included in diet and not engaged in lipogenesis in favour of tumor, however, used in this process due to energy demand, of which they may be the source [65, 76, 77]; on the other hand, they announced lipogenesis promotion in favour of tumor and limitation the anti-cancerous activity [78].

## Conclusions

In conclusion, the results point to ATME as the microenvironment which is extremely active in lipid metabolism accompanying SCC of the oral cavity. This is reflected by high content of certain FAs mainly in ATME and decrease in frequency of some other FAs in tumor in comparison with ATME. ATME was the environment in which the greatest number of FAs showed differences in percentage content between G1+2 and G3 tumors and these differences were the largest. Primary role of tumor grade in these changes was revealed mainly in ATME although in all three environments, i.e. tumor, ATME and serum, tumor grade influenced the number and magnitude of associations between particular FAs.

Moreover, significant differences in FAs content between G1+2 vs G3 SCCs suggest that different FAs may play a role in metabolism of lower (G1+G2) and higher grade (G3) tumors and high content and frequency of certain FAs in grade 3 tumors suggest FAs significance in the process of tumor progression. The results show that relation between percentage of certain FAs and tumor grade may be positive or negative depending on the microenvironment. Finally, correlations between percentage content of particular FAs within the same microenvironment differed depending on tumor grade. The correlations suggest that in tumors grade 3 lipogenesis in tumor tissue coexisted with inflammation and in tumors grade 1+2 lipogenesis was modulated by EPA.

## Supporting information

S1 Table Percentage of FAs content in tumor, ATME, and blood serum *vs*. tumor grade. (DOCX)

S2 Table Percentage of content of FAs groups: SFA, UFA, MUFA, series n3 of PUFA and ratios C18:2n6/C18:3n3, C20:4/(C20:5+C22:6n3) in tumor, ATME and blood serum *vs*. tumor grade. (DOCX)

S3 Table Ratio of percentage content of particular FAs in tumor/ATME, ATME/serum and in tumor/serum vs. tumor grade. (DOCX)

## References

[1] Hanahan D, Weinberg RA. The hallmarks of cancer. The Next Generation. Cell. 2011 3 4;144(5):646–74. 10.1016/j.cell.2011.02.013 21376230

[2] Jone SF, Infante JR. Molecular pathways: fatty acid synthase. Clin Cancer Res. 2015 12 15;21(24):5434–8. 10.1158/1078-0432.CCR-15-0126 26519059

[3] Louie SM, Roberts LS, Mulvihill MM, Luo K, Nomura DK. Cancer cells incorporate and remodel exogenous palmitate into structural and oncogenic signalling lipids. Biochim. Biopfys Acta. 2013 10;1831(10):1566–72. 10.1016/j.bbalip.2013.07.008 PMC3838875 23872477

[4] Agostini M, Silva SD, Zecchin KG, Coletta RD, Jorge J, Loda M et al Fatty acid synthase is required for the proliferation of human oral squamous carcinoma cells. Oral Oncol. 2004 8;40(7):728–35. 10.1016/j.oraloncology.2004.01.011 15172643

[5] Guo C, Cui L, Yu G, Liu D, Meng S, Qing Song Q. Endogenous fatty acid synthesis in squamous cell carcinomas of the oral cavity. Brit Jour of Oral and Maxillofacial Surg. 2000 10;38(5):506–508. 10.1054/bjom.2000.0311 11010783

[6] Menendez JA, Lupu R: Oncogenic properties of the endogenous fatty acid metabolism: molecular pathology of fatty acid synthase in cancer cells. Curr Opin Clin Nutr Metab Care. 2006 7;9(4):346–57. 10.1097/01.mco.0000232893.21050.15 16778562

[7] Witz IP. The tumor microenvironment: the making of a paradigm. Cancer Microenviron. 2009 9;2 Suppl 1:9–17. 10.1007/s12307-009-0025-8 PMC2756342 19701697

[8] Koontongkaew S. The tumor microenvironment contribution to development, growth, invasion and metastasis of head and neck squamous cell carcinomas. J Cancer. 2013; 4(1): 66–83. 10.7150/jca.5112 23386906 PMC3564248

[9] Troester MA, Lee MH, Carterd M, Fan C, Cowan DW, Perez ER. Activation of host wound responses in breast cancer microenvironment. Clin Cancer Res. 2009 11 15; 15(22): 7020–7028. 10.1158/1078-0432.CCR-09-1126 19887484 PMC2783932

[10] Casbas-Hernandez P, Sun X, Roman-Perez E, D’rcy M, Sandhu MR, Hishida A et al Tumor intrinsic subtype is reflected in cancer-adjacent tissue. Cancer Epidemiol Biomarkers Prev. 2015 2 24(2): 406–414. 10.1158/1055-9965.EPI-14-0934 25465802 PMC4437571

[11] Folch J, Lees M, Sloane SGH. A simple method for the isolation and purification of total lipides from animal tissues. J Biol Chem. 1957;226: 497–509. 13428781

[12] Dallal GE. Obtaining superscripts to affix to means that are not significantly different from each other. 2000. www.jerrydallal.com/LHSP/similar_prog.htm.

[13] Friendly M. Corrgrams: exploratory displays for correlation matrices. American Statistician. 2002;56: 316–324.

[14] Currie E, Schulze A, Zechner R, Tobias C, Walther TC, Farese RV Jr. Cellular Fatty acid metabolism and cancer. Cell Metab. 2013 8 6;18(2):153–61. 10.1016/j.cmet.2013.05.017 23791484 PMC3742569

[15] Beloribi-Djefaflia S, Vasseur S, Guillaumond F. Lipid metabolic reprogramming in cancer cells. Oncogenesis. (2016) 5, e189 10.1038/oncsis.2015.49 26807644 PMC4728678

[16] Peck B, and Schulze A. Lipid desaturation–the next step in targeting lipogenesis in cancer? FEBS Journal 2016, 283, 2767–2778. 10.1111/febs.13681 26881388

[17] Ray U, and Roy SS. Aberrant lipid metabolism in cancer cells–the role of oncolipid- activated signaling. FEBS Journal 2018; 285, 432–443. 10.1111/febs.14281 28971574

[18] Li S, Zhou T, Li C, Dai Z, Che D, Yao Y, et al (2014) High Metastaticgastric and Breast Cancer Cells Consume Oleic Acid in an AMPK Dependent Manner. PLoS ONE 2014 5 9; (5): e97330 10.1371/journal.pone.0097330. 24823908 PMC4019637

[19] Kuroki S, Yokoo S, Terashi H, Hasegawa M, Komori T. Epithelialization in Oral Mucous Wound Healing in Terms of Energy Metabolism Kobe J. Med. Sci.2009, Vol. 55, No. 2, pp. E5–E15. 19628973

[20] Askari M, Darabi M, Zare R, Mahmudabadi, Obodiat M, Fayezi S et al Tissue fatty acid composition and secretory phospholipase-A2 activity in oral squamous cell carcinoma. Clin Transl Oncol. 2015;17(5): 378–83. 10.1007/s12094-014-1242-2 25351172

[21] Menendez JA, Colomerc R, Lupu R. Why does tumor-associated fatty acid synthase (oncogenic antigen-519) ignore dietary fatty acids? Medical Hypotheses. 2005;64(2): 342–349. 10.1016/j.mehy.2004.07.022 15607569

[22] Menendez JA, Lupu R. Fatty acid synthase and the lipogenic phenotype in cancer pathogenesis. Nature Reviews Cancer. 2007;7: 763–777. 10.1038/nrc2222 17882277

[23] Menendez JA, Vellon L, Espinoza I, Lupu R. The metastasis inducer CCN1 (CYR 6 1) activates the fatty acid synthase (FASN)–driven lipogenic phenotype in breast cancer cells. Oncoscience. 2016 7;3,(7–8):242–257. 10.18632/oncoscience.314 27713913 PMC5043073

[24] Kaur G, Cameron-Smith D, Garg M, Sinclair AJ. Docosapentaenoic acid (22:5n-3): A review of its biological effect. Prog Lipid Res. 2011 1;50,(1):28–34. 10.1016/j.plipres.2010.07.004 20655949

[25] Sprecher H. Metabolism of highly unsaturated n-3 and n-6 fatty acids. Biochimica et Biophysica Acta. 2000 7; 1486(2–3):219–231. 10.1016/s1388-1981(00)00077-9 10903473

[26] Berquin IM, Edwards IJ, Chen YQ. Multi-targeted therapy of cancer by omega-3 fatty acids. Cancer Lett. 2008 10 8;269(2):363–377. 10.1016/j.canlet.2008.03.044 18479809 PMC2572135

[27] Arterburn LM, Hall EB, Oken H. Distribution, interconversion, and dose response of n_3 fatty acids in humans. Am J Clin Nutr. 2006 6;83(6 suppl): 1467S–1476S. 10.1093/ajcn/83.6.1467S 16841856

[28] Yazdi PG. A review of the biologic and pharmacologic role of docosapentaenoic acid n-3 [version 2; referees: 2 approved]. F1000Research 2014; 2:256 (10.12688/f1000research.2-256.v2) PMC4162505 25232466

[29] Tsuji M, Murota SI, Morita I. Docosapentaenoic acid (22:5, n-3) suppressed tube- forming activity in endothelial cells induced by vascular endothelial growth factor. Prostaglandins Leukot Essent Fatty Acids. 2003 5; 68(5):337–342. 12711251 10.1016/s0952-3278(03)00025-5

[30] Morin C, Rousseau E, Fortin S. Anti-proliferative effects of a new docosapentaenoic acid monoacylglyceride in colorectal carcinoma cells. PLEFA. 2013 9; 89 (4): 203–213. 10.1016/j.plefa.2013.07.004 23932824

[31] Bektas-Kayhan K. Role of Inflammation in Oral Squamous Cell Carcinoma. ISBN: 978-953-51-0024-9. InTech. 2012. http://www.intechopen.com/books/squamous-cell-carcinoma/role-of-inflammation-in-oral-squamous-cellcarcinoma.

[32] Hecker M, Sommer N, Voigtmann H, Pak O, Mohr A, Wolf M. Impact of short- and medium-chain fatty acids on mitochondrial function in severe inflammation. JPEN J Parenter Enteral Nutr. 2014 7; 38(5): 587–94. 10.1177/0148607113489833 23703093

[33] Kopf PG, Zhang DX, Gauthier KM, Nithipatikom K, Yi XY, Falck JR et al Adrenic acid metabolites as endogenous endothelium-derived and zona glomerulosa-derived hyperpolarizing factors. Hypertension. 2010 2;55(2):547–54 10.1161/HYPERTENSIONAHA.109.144147 20038752 PMC2819927

[34] Ghebremeskel K, Min Y, Crawford MA, Nam JH, Kim A, Koo JN et al Blood fatty acid composition of pregnant and nonpregnant Korean women: red cells may act as a reservoir of arachidonic acid and docosahexaenoic acid for utilization by the developing fetus. Lipids.2000 5;35(5):567–74. 10907792 10.1007/s11745-000-557-3

[35] Burrage LC, Miller MJ, Wong LJ, Kennedy AD, Sutton VR,Qin Sun. Elevations of C14:1 and C14:2 plasma acylcarnitines in fasted children: a diagnostic dilemma. J Pediatrics. 2015 11;169:208–13.e 2. 10.1016/j.jpeds.2015.10.045 PMC4729603 26602010

[36] Cater NB, Denke MA. Behenic acid is a cholesterol-raising saturated fatty acid in humans. Am J Clin Nutr. 2001 1;73(1):41–4. 10.1093/ajcn/73.1.41 11124748

[37] Kritchevsky SB, Kritchevsky D. Serum cholesterol and cancer risk: an epidemiologic perspective. Annu Rev Nutr. 1992;12: 391–416. 10.1146/annurev.nu.12.070192.002135 1503812

[38] Liu J, Ma DWL. The Role of *n*-3 Polyunsaturated Fatty Acids in the Prevention and Treatment of Breast Cancer. Nutrients. 2014 11 18;6(11): 5184–5223. 10.3390/nu6115184 25412153 PMC4245586

[39] Yang P, Jiang Y, Fischer SM. Prostaglandin E_3_ metabolism and cancer. Cancer lett. 2014 6 28;348(0): 1–11. 24657656 10.1016/j.canlet.2014.03.010 PMC4366418

[40] D’Eliseo D, Velotti F. Omega-3 fatty Acids and cancer cell cytotoxicity: implications for multi-targeted cancer therapy. Brown L, Rauch B, Poudyal H, eds. J Clin Med. 2016 2; 5(2): 15 10.3390/jcm5020015. PMC4773771 26821053

[41] Saadatian-Elahi M, Norat T, Goudable J, Riboli E. Biomarkers of dietary fatty acid intake and the risk of breast cancer: A meta-analysis. Int. J. Cancer. 2004 9;111 (4): 584–591. 10.1002/ijc.20284. 15239137

[42] Lohe VK, Degwekar SS, Bhowate RR, Kadu RP, Dangore SB. Evaluation of correlation of serum lipid profile in patients with oral cancer and precancer and its association with tobacco abuse. J Oral Pathol Med. 2010 2;39,(2): 141–148. 10.1111/j.1600-0714.2009.00828.x 20002982

[43] Singh S, Ramesh V, Premalatha B, Prashad KV, Ramadoss K. Alterations in serum lipid profile patterns in oral cancer. J Nat Sc Biol Med 2013;4:374–8. 24082735 10.4103/0976-9668.116994 PMC3783783

[44] Kumar P, Augustine J, Urs AB, Arora S, Gupta S, Mohanty VR. Serum lipid profile in oral cancer and leukoplakia: Correlation with tobacco abuse and histological grading. J Can Res Ther. 2012;8(3): 384–8. 10.4103/0973-1482.103517 23174719

[45] Sherubin E.J., Kannan K.S., Kumar D.N., Joseph I..: Estimation of plasma lipids and its significance on histopathological grades in oral cancer: Prognostic significance an original research. J Oral Maxillofac Pathol. 2013 Jan-Apr; 17(1): 4–9. 10.4103/0973-029X.110685 23798822 PMC3687186

[46] Abraham AR, Bahl VK, Parshad R, Seenu V, Roy V, Golandaz S. Content of Trans Fatty Acids in Human Cheek Epithelium: Comparison with Serum and Adipose Tissue. BioMed Research International Volume 2013, Article ID 276174, 7 pages. 10.1155/2013/276174 PMC3816018 24222900

[47] Zhao J, Lyu C, Gao J, Du L, Shan B, Zhang H et al Dietary fat intake and endometrial cancer risk: A dose response meta-analysis. Medicine (Baltimore). 2016 7;95(27):e4121. 27399120 10.1097/MD.0000000000004121 PMC5058849

[48] Basset J.K., Hodge A.M., English D.R., Mac Innis R.J., Giles G.G.: Plasma phospholipids fatty acids, dietary fatty acids, and breast cancer risk. Cancer Causes Control. 2016 6; 27(6):759–73. 10.1007/s10552-016-0753-2 27146840

[49] Mansara PP, Deshpande RA, Vaidya MM, Kaul-Ghanekar R. Differential ratios of omega fatty acids (AA/EPA+DHA) modulate growth, lipid peroxidation and expression of tumor regulatory MARBPs in breast cancer cell lines MCF7 and MDA-MB-231. PLoS ONE. 2015;10(9). 10.1371/journal.pone.0136542 PMC4556657 26325577

[50] Crowe FL, Allen NE, Appleby PN, Overvad K, Aardestrup IV, Johnsen NF. Fatty acid composition of plasma phospholipids and risk of prostate cancer in a case- control analysis nested within the European Prospective Investigation into Cancer and Nutrition. Am J Clin Nutr. 2008 11;88 (5): 1353–63. 10.3945/ajcn.2008.26369 18996872

[51] Ritch CR, Wan RL, Stephens LB, Taxy JB, Huo D, Gong EM et al Dietary fatty acids correlate with prostate cancer biopsy grade and volume in Jamaican men. J Urol. 2007 1;177(1): 97–101. 10.1016/j.juro.2006.08.105 17162011

[52] Khodarhmi M, Azadbakht L. The association between different kinds of fat intake and breast cancer risk in women. Int J Prev Med. 2014 1;5(1): 6–15. 24554986 PMC3915474

[53] Zhang P, Wen X, Gu F, Zhang X, Li J, Liu Y, Dong J, Deng X, Zhu X, Tian Y. Role of serum polyunsaturated fatty acids in the development of colorectal cancer. International J Clin Exper Med. 2015 9;8(9):15900–15909. PMC4658982 26629093

[54] Baracos VE, Mazurak VC, Ma DW. n-3 Polyunsaturated fatty acids throughout the cancer trajectory: influence on disease incidence, progression, response to therapy and cancer-associated cachexia. Nutr Res Rev. 2004 12;17(2): 177–92. 10.1079/NRR200488 19079925

[55] Ravagnan L, Roumier T, Kroemer G. Mitochondria, the killer organelles and their weapons. J Cell Physiol. 2002 8;192(2): 131–7. 10.1002/jcp.10111 12115719

[56] Palakurthi SS, Flückiger R, Aktas H, Changolkar AK, Shahsafaei A, Harneit S et al Inhibition of translation initiation mediates the anticancer effect of the n-3 polyunsaturated fatty acid eicosapentaenoic acid. Cancer Res. 2000 6 1;60(11):2919–25. 10850438

[57] Barber MD, Fearon KC, Tisdale MJ, McMillan DC, Ross JA. Effect of a fish oil- enriched nutritional supplement on metabolic mediators in patients with pancreatic cancer cachexia. Nutr Cancer. 2001;40(2): 118–24. 10.1207/S15327914NC402_7 11962246

[58] Rose DP, Connolly JM. Antiangiogenicity of docosahexaenoic acid and its role in the suppression of breast cancer cell growth in nude mice. Int J Oncol. 1999;15: 1011–1015. 10.3892/ijo.15.5.1011 10536186

[59] Iwamoto S, Senzaki H, Kiyozuka Y, Ogura E, Takada H, Hioki K, Tsubura A. Effects of fatty acids on liver metastasis of ACL-15 colon cancer cells. Nutr Canc. 1998;31(2):143–150. 10.1080/01635589809514694 9770727

[60] Pratt VC, Watanabe S, Bruera E, Mackey J, Clandinin MT, Baracos VE et al Plasma and neutrophil fatty acid composition in advanced cancer patients and response to fish oil supplementation. Br J Cancer. 2002 12 2;87(12): 1370–8. 10.1038/sj.bjc.6600659 12454764 PMC2376285

[61] Aggarwal BB, Gehlot P. Inflammation and cancer:how friendly is the relationship for cancer patients? Curr Opin Pharmacol. 2009 8;9(4): 351–369. 10.1016/j.coph.2009.06.020 19665429 PMC2730981

[62] Calder PC. The role of marine omega-3 (n-3) fatty acids in inflammatory processes, atherosclerosis and plaque stability. Mol Nutr Food Res. 2012 7;56(7): 1073–1080. 10.1002/mnfr.201100710 22760980

[63] Biswas NK, Das S, Maitra A, Sarin R, Majumder PP. Somatic mutations in arachidonic acid metabolism pathway genes enhance oral cancer post-treatment disease-free survival. Nat Comm.5 2014; 5835. 10.1038/ncomms6835 25517499

[64] Piva MR, DE Souza LB, Martins-Filho PR, Calazans Soares R, DE Santana Santos T, DE Souza Andrade ES et al Role of inflammation in oral carcinogenesis (Part I): Histological grading of malignancy using a binary system. Oncol Lett. 2011 11;2(6): 1225–1231. 10.3892/ol.2011.382 22848292 PMC3406493

[65] Schönfeld P, Wojtczak L. Short- and medium-chain fatty acids in energy metabolism: the cellular perspective. The Journal of Lipid Research. 2016 6;57(6): 943–954. 10.1194/jlr.R067629 27080715 PMC4878196

[66] Zhang G, Panigrahy D, Mahakian LM, Yang J, Liu JY, Stephen Lee KS et al Epoxy metabolites of docosahexaenoic acid (DHA) inhibit angiogenesis, tumor growth, and metastasis. Proc Natl Acad Sci U S A. 2013 4 16;110(16):6530–6535. 10.1073/pnas.1304321110 23553837 PMC3631682

[67] Iijima H, Kasai N, Chiku H, Murakami S, Sugawara H, Sakaguchi K et al The inhibitory action of long-chain fatty acids on the DNA binding activity of p53. Lipids. 2006; 41,6: 521–527. 10.1007/s11745-006-5000-2. 16981429

[68] Azrad M, Turgeon C, Demark-Wahnefried W. Current evidence linking polyunsaturated Fatty acids with cancer risk and progression. Front Oncol. 2013 9 4;3: 224 10.3389/fonc.2013.00224 24027672 PMC3761560

[69] Kim J, Park HD, Park E, Chon J, Park YK. Growth‐inhibitory and proapoptotic effects of alpha‐linolenic acid on estrogen‐positive breast cancer cells. Ann N Y Acad Sci. 2009 8;1171:190–5. 10.1111/j.1749-6632.2009.04897.x 19723055

[70] Frigolet ME, Gutiérrez-Aguilar R. The role of the novel lipokine palmitoleic acid in health and disease. Advances in Nutrition. 2017 1 17;8(1):173S–181S. 10.3945/an.115.011130 28096141 PMC5227969

[71] Hess D, Chisholm JW, Igal RA. Inhibition of StearoylCoA Desaturase Activity Blocks Cell Cycle Progression and Induces Programmed Cell Death in Lung Cancer Cells. PLoS ONE. 2010;5(6): e11394 10.1371/journal.pone.0011394 20613975 PMC2894866

[72] Jiang L, Wang W, He Q, Wu Y, Lu Z, Sun J. Oleic acid induces apoptosis and autophagy in the treatment of Tongue Squamous cell carcinomas. Sci Rep.2017 9 12;7(1):11277. Article number: 11277 (2017). 28900281 10.1038/s41598-017-11842-5 PMC5595908

[73] Calder PC Omega-3 fatty acids and inflammatory processes. Nutrients. 2010 3;2: 355–374. 10.3390/nu2030355 22254027 PMC3257651

[74] Nikolakopoulou Z, Nteliopoulos G, Michael-Titus AT, Parkinson EK. Omega-3 polyunsaturated fatty acids selectively inhibit growth in neoplastic oral keratinocytes by differentially activating ERK1/2. Carcinogenesis. 2013 12;34,(12): 2716–2725. 10.1093/carcin/bgt257 23892603 PMC3845892

[75] Pappalardo G, Almeida A, Ravasco P. Eicosapentaenoic acid in cancer improves body composition and modulates metabolism. Nutrition. 2015 4;31(4):549–55. 10.1016/j.nut.2014.12.002 25770317

[76] Risérus U, and Marklund M. Milk fat biomarkers and cardiometabolic Disease. Curr Opin Lipidol 2017, 28:46–51. 10.1097/MOL.0000000000000381 27906713 PMC5214382

[77] Hu J, La Vecchia C, de Groh M, Negri E, Morrison H, Mery L. Canadian Cancer Registries Epidemiology Research Group: Dietary trans fatty acids and cancer risk. Eur J Cancer Prev. 2011 11;20(6):530–8. 10.1097/CEJ.0b013e328348fbfb 21701388

[78] Jiang WG, Bryce RP, Horrobin DF. Essential fatty acids: molecular and cellular basis of their anti-cancer action and clinical implications. Critical Reviews in Oncology / Hematology. 1998;27,3: 179–209. 9649932 10.1016/s1040-8428(98)00003-1

