# Supplementary material for: Fatty acids distribution and content in oral squamous cell carcinoma tissue and its adjacent microenvironment
Source: PLoS One. 2019 Jun 26;14(6):e0218246. doi: 10.1371/journal.pone.0218246 (PMC6594603; doi:10.1371/journal.pone.0218246)
Supplement: S1 Table — (DOCX) [file pone.0218246.s001.docx]

**S1 Table.** Percentage of FAs content in tumor, ATME, and blood serum *vs.* tumor grade.

| **FAs** | **Mean % in tumor** | | **Mean % in ATME** | | **Mean % in serum** | |
| --- | --- | --- | --- | --- | --- | --- |
|  | **(G1+G2)** | **(G3)** | **(G1+G2)** | **(G3)** | **(G1+G2)** | **(G3)** |
| **C10:0** | 0.76±0.77 | 0.0±0.000* | 0.24±0.52 | 0.00±0.00* | 0.12±0.15 | 0.26±0.04* |
| **C 12:0** | 0.17±0.17 | 0.00±0.01* | 0.23±0.18 | 0.01±0.02* | 0.41±0.16 | 0.41±0.17 |
| **C18:0** | 16.77±8.70 | 24.66±9.25 | 8.51±8.58 | 20.09±12.24* | 20.12±3.29 | 19.72±1.81 |
| **C18:1n9** | 23.10±9.36 | 15.80±9.25 | 38.12±12.69 | 24.85±13.62* | 16.91±3.31 | 15.40±2.15 |
| **C18:2n6** | 9.62±3.27 | 8.95±2.29 | 10.56±2.79 | 8.00±1.97* | 11.94±1.74 | 12.56±2.73 |
| **C18:3n-3** | 0.31±0.32 | 0.17±0.24 | 0.81±0.42 | 0.27±0.32* | 0.38±0.26 | 0.60±0.33 |
| **C20:5** | 0.25±0.14 | 0.46±0.39 | 0.15±0.12 | 0.22±0.13 | 0.41±0.32 | 0.66±0.19* |
| **C22:0** | 0.23±0.21 | 0.48±0.45 | 0.12±0.24 | 0.25±0.12* | 0.00±0.00 | 0.00±0.00 |
| **C22:1n13** | 0.28±0.23 | 0.27±0.24 | 0.12±0.16 | 0.35±0.43* | 0.00±0.00 | 0.00±0.00 |
| **C23:0** | 0.39±0.57 | 1.09±0.45* | 0.32±0.52 | 1.00±0.60* | 0.00±0.00 | 0.00±0.00 |
| **C22:6n3** | 1.64±0.66 | 1.90±0.78 | 0.62±0.45 | 1.28±0.89* | 1.45±0.38 | 1.73±0.30 |

⃰ p < 0,05

G – grade
