# Supplementary material for: Fatty acids distribution and content in oral squamous cell carcinoma tissue and its adjacent microenvironment
Source: PLoS One. 2019 Jun 26;14(6):e0218246. doi: 10.1371/journal.pone.0218246 (PMC6594603; doi:10.1371/journal.pone.0218246)
Supplement: S2 Table — (DOCX) [file pone.0218246.s002.docx]

**S2 Table.** Percentage of content of FAs groups: SFA, UFA, MUFA, series n3 of PUFA and ratios C18:2n6/C18:3n3, C20:4/(C20:5+C22:6n3) in tumor, ATME and blood serum *vs.* tumor grade

| **Groups of FAs** | **Mean % in tumor** | | **Mean % in ATME** | | **Mean % in serum** | |
| --- | --- | --- | --- | --- | --- | --- |
|  | **(G1+G2)** | **(G3)** | **(G1+G2)** | **(G3)** | **(G1+G2)** | **(G3)** |
| **SFA** | 47.22±11.51 | 58.53±14.06 | **36.67±13.48** | **52.80±16.23*** | 61.05±5.71 | 60.8±3.262 |
| **MUFA** | 31.41±11.03 | 21.85±11.55 | **48.03±14.74** | **32.07±17.92*** | 20.18±4.13 | 18.48±2.68 |
| **n3** | 3.23±1.13 | 3.62±1.39 | 2.17±0.64 | 2.48±1.25 | **2.30±0.74** | **3.24±0.57*** |
| **n6/n3** | 5.77±1.30 | 4.69±1.01 | 6.24±1.18 | 6.12±2.63 | **7.68±1.98** | **5.55±1.35*** |
| **C20:4/(C20:5+C22:6n3)** | **3.74±0.97** | **2.95±0.65*** | 2.57±0.93 | 2.63±0.63 | 2.40±0.72 | 1.89±0.46 |
| **UFA** | 52.78±11.51 | 41.47±14.06 | **63.33±13.48** | **47.20±16.23*** | 38.95±5.71 | 39.18±3.26 |
| **PUFA/SFA** | 0.48±0.16 | 0.37±0.18 | **0.45±0.13** | **0.31±0.11*** | 0.31±0.07 | 0.34±0.07 |
| **MUFA/SFA** | 0.75±0.43 | 0.45±0.36 | **1.55±0.71** | **0.77±0.63*** | 0.34±0.10 | 0.31±0.05 |
| **UFA/SFA** | 1.23±0.50 | 0.82±0.53 | **2.00±0.80** | **1.08±0.71*** | 0.65±0.16 | 0.65±0.09 |

p < 0,05

G – grade
