# Supplementary material for: Fatty acids distribution and content in oral squamous cell carcinoma tissue and its adjacent microenvironment
Source: PLoS One. 2019 Jun 26;14(6):e0218246. doi: 10.1371/journal.pone.0218246 (PMC6594603; doi:10.1371/journal.pone.0218246)
Supplement: S3 Table — (DOCX) [file pone.0218246.s003.docx]

**S3 Table**. Ratio of percentage content of particular FAs in tumor/ATME, ATME/serum and in tumor/serum vs. tumor grade.

| **FAs** | **G1+G2** | | | **G3** | | |
| --- | --- | --- | --- | --- | --- | --- |
|  | **tumor/**  **ATME** | **tumor/**  **serum** | **ATME/**  **serum** | **tumor/**  **ATME** | **tumor/**  **serum** | **ATME/**  **serum** |
| **C10:0** | 3.13 | 6.49 | 2.07 | 0.00 | 0.00 | 0.00 |
| **C12:0** | 0.74 | 0.43 | 0.58 | 0.31 | 0.01 | 0.03 |
| **C13:0** | 0.00 | 0.00 | 0.00 | 0.00 | 0.00 | 0.00 |
| **C14:0** | 1.07 | 1.49 | 1.39 | 0.63 | 1.26 | 1.99 |
| **C14:1** | 0.66 | 27.25 | 41.31 | 0.47 | 4.93 | 10.49 |
| **C15:0** | 1.56 | 0.37 | 0.23 | 0.94 | 0.27 | 0.28 |
| **C16:0** | 1.05 | 0.70 | 0.67 | 1.07 | 0.80 | 0.75 |
| **C16:1** | 0.55 | 2.19 | 3.98 | 0.60 | 1.55 | 2.57 |
| **C17:0** | 1.98 | 0.80 | 0.40 | 0.81 | 0.75 | 0.92 |
| **C18:0** | 1.97 | 0.83 | 0.42 | 1.23 | 1.25 | 1.02 |
| **C18:1n9** | 0.61 | 1.37 | 2.25 | 0.64 | 1.03 | 1.61 |
| **C18:1trans11** | 0.92 | 1.71 | 1.85 | 1.29 | 1.82 | 1.41 |
| **C18:2n6** | 0.91 | 0.81 | 0.88 | 1.12 | 0.71 | 0.64 |
| **C18:3n-6** | 4.85 | 0.11 | 0.02 | 0.00 | 0.00 | 0.05 |
| **C18:3n-3** | 0.38 | 0.80 | 2.13 | 0.63 | 0.28 | 0.45 |
| **C18:4** | 0.74 | 0.00 | 0.00 | 0.00 | 0.00 | 0.00 |
| **C20:4** | 3.29 | 1.73 | 0.53 | 1.57 | 1.49 | 0.95 |
| **C20:5** | 1.63 | 0.60 | 0.37 | 2.11 | 0.70 | 0.33 |
| **C22:0** | 2.01 | 0.00 | 0.00 | 1.95 | 0.00 | 0.00 |
| **C22:1n13** | 2.34 | 0.00 | 0.00 | 0.77 | 0.00 | 0.00 |
| **C23:0** | 1.20 | 0.00 | 0.00 | 1.09 | 0.00 | 0.00 |
| **C22:4n6** | 3.43 | 86.10 | 25.09 | 1.03 | 0.00 | 0.00 |
| **C22:5n3** | 1.89 | 17.95 | 9.50 | 1.51 | 4.27 | 2.83 |
| **C22:6n3** | 2.63 | 1.13 | 0.43 | 1.48 | 1.09 | 0.74 |
| **C24:1** | 5.79 | 0.00 | 0.00 | 0.77 | 0.00 | 0.00 |

G - grade
